# Human Mannose Receptor 1 Attenuates HIV-1 Infectivity in a Virus Isolate-Specific Manner

**DOI:** 10.3390/v15102057

**Published:** 2023-10-06

**Authors:** Hideki Saito, Sayaka Sukegawa, Sandra Kao, Klaus Strebel

**Affiliations:** Viral Biochemistry Section, Laboratory of Molecular Microbiology, NIAID, NIH, Bethesda, MD 20892, USA; hideki.saito@nih.gov (H.S.); sukemolv@tmd.ac.jp (S.S.); skao@niaid.nih.gov (S.K.)

**Keywords:** HIV-1, mannose receptor, restriction factor, virus host interactions, virus assembly

## Abstract

Human mannose receptor 1 (hMRC1) is a transmembrane glycoprotein that belongs to the C-type lectin family and is expressed on the surface of most tissue macrophages. hMRC1 contributes to the binding and transmission of HIV-1 and is involved in the endocytic uptake of HIV-1 for subsequent antigen presentation. We previously reported that hMRC1 functions as an antiviral factor by inhibiting virus release through a BST-2-like mechanism. The inhibition of virus release was not virus isolate-specific and, surprisingly, was not Env-dependent. We now report on another hMRC1 antiviral function that affects the infectivity of viral particles. Unlike its effect on virus release, the inhibition of viral infectivity by hMRC1 was virus isolate-specific. An analysis of chimeric Env revealed that the Env V3 region was a critical determinant for the inhibitory effect of hMRC1. Of note, exogenously expressed hMRC1 was packaged into viral particles in an Env-independent manner. Co-immunoprecipitation studies revealed a strong interaction of the hMRC1-sensitive NL43 Env with hMRC1, while the hMRC1-insensitive Envs of AD8 and 49.5 isolates interacted poorly if at all with hMRC1. An analysis of a panel of Transmitted/Founder (T/F) viruses revealed that all of them were R5-tropic, and more than half of them were inhibited by hMRC1. The detailed mechanism of how hMRC1 inhibits viral infectivity remains to be investigated. However, the high-affinity binding of hMRC1 to Env may cause a conformational change around the Env V3 region or obstruct the Env V3 region and may make it inaccessible for subsequent interaction with the coreceptor during virus entry.

## 1. Introduction

Human mannose receptor C-type 1 (hMRC1), also known as macrophage mannose receptor (MMR) or CD206, is a 175 kDa single-pass transmembrane glycoprotein that belongs to the C-type lectin family and is expressed on the surface of most tissue macrophages, dendritic cells (DCs), and some lymphatic or liver endothelial cells (reviewed in [1]). The expression levels of MMR on macrophages are estimated to reach upwards of 100,000 molecules per cell [2]. The protein consists of an N-terminal cysteine-rich domain, a fibronectin type II repeat, and eight carbohydrate recognition domains (reviewed in [3]). MRC1 was reported to serve as an entry receptor for invading pathogens, such as bacteria, fungi, viruses (incl. HIV-1), and other parasites (reviewed in [1]). Of note, the hMRC1-mediated uptake of HIV-1 by macrophages does not lead to productive infection [4,5]. Instead, the mannose receptor-mediated uptake of HIV-1, Dengue virus, HBV, or Influenza A virus results in the processing of pathogens in MHC-II-containing compartments for subsequent antigen presentation [3,6,7,8]. Thus, MRC1 constitutes part of the cellular innate host defense mechanism. However, some pathogens, including HIV-1, have learned to antagonize the innate immune function of MRC1 by downregulating the mannose receptor from the cell surface [9,10,11,12].

Previous studies have implicated the viral Tat, Nef, and Vpr proteins in MRC1 downmodulation, although the relative contribution of each of these factors remains under debate [13,14,15]. Among those, Nef is thought to promote the internalization of MRC1 from the cell surface without affecting cellular steady-state levels [15]. As far as Vpr is concerned, one recent study identified a role of Vpr in the downmodulation of hMRC1 at the total cellular protein level [16]. The authors reported that the expression of Vpr reduced hMRC1 steady-state levels through a mechanism that required an interaction with DCAF1 but did not involve the degradation of hMRC1 via the direct proteasomal pathway that Vpr normally uses to degrade other substrates, such as UNG2 [16]. However, our own investigation of HIV-1-infected macrophages did not reveal a significant contribution of any of the viral accessory proteins. Indeed, the inactivation of *vif*, *vpu*, *nef*, or *vpr* genes alone when studied in the context of the macrophage-tropic AD8 full-length infectious molecular clone did not abolish its ability to inhibit hMRC1 expression at the total protein level [17]. Thus, if viral accessory proteins are involved in downmodulating hMRC1 at the protein level, it would have to be through a concerted effort of multiple accessory proteins, for which there is, however, no experimental evidence.

HIV-1 Tat was found to inhibit MRC1 expression at the transcriptional level [14,17,18]. The inhibitory effect of Tat on MRC1 expression was not direct but involved PU.1, a myeloid cell-specific transcription factor involved in the cellular regulation of MRC1 transcription. Indeed, we identified a complex interplay between HIV-1 Tat and PU.1, which upregulates the MRC1 promoter. Interestingly, while Tat activates HIV-1 gene expression through a positive feedback loop, PU.1 is involved in a negative feedback loop that acts on the HIV-1 LTR promoter and inhibits viral gene expression, including Tat. Tat, however, inhibits the activity of PU.1, thereby antagonizing the inhibitory effect of PU.1 on LTR-based transcription. Thus, we found that there is a complex equilibrium between viral and host gene expression in HIV-infected macrophages [17].

Our current study aimed to investigate the importance of hMRC1 downmodulation in HIV-1 replication. We found that, in addition to inhibiting the release of progeny virions from HIV-1-infected cells, the expression of hMRC1 also affected viral infectivity. Interestingly, while the inhibition of viral particle release was a generic feature of hMRC1 affecting all virus isolates tested, the effect of hMRC1 on virus infectivity was virus isolate-specific. Thus, the infectivity of the X4-tropic NL43 isolate was inhibited by hMRC1 overexpression, while the infectivity of the R5-tropic isolates AD8 and 49.5 was not affected. However, sensitivity to hMRC1 turned out to not be related to viral tropism since the infectivity of more than half of a panel of R5-tropic Transmitted/Founder (T/F) viruses was sensitive to hMRC1. Nevertheless, an analysis of Env chimeras revealed an important contribution of the Env V3 loop known for its importance in determining cell tropism (reviewed in [19]). Interestingly, we found that NL43 Env but not the Env of the V3 loop variant 49.5 or the R5-tropic AD8 interacted with hMRC1 intracellularly. Furthermore, we found that hMRC1, when overexpressed, was packaged into cell-free virions, where it interacted with the NL43 Env protein but not with the R5-tropic 49.5 variant or AD8. Our results suggest that hMRC1, when packaged into virions, can reduce viral infectivity by interfering with the normal function of certain Env proteins, such as mediating coreceptor interactions during infection.

## 2. Materials and Methods

### 2.1. Cells

HEK293T and TZM-bl cells were maintained in Dulbecco’s Modified Eagle’s Medium (DMEM) with 4.5 g/L glucose (Lonza, Walkersville, MD, USA), supplemented with 10% fetal bovine serum, 100 U/mL penicillin, and 100 µg/mL streptomycin in a 37 °C and 5.0% CO_2_ environment.

### 2.2. Plasmid and Viral Vectors

The construction of the hMRC1 expression plasmid pCMV6-hMRC1 has been reported previously [18]. A variant carrying a C-terminal 3xHA epitope tag was created via the PCR amplification of pCMV6-hMRC1 with primers creating a 5′ NheI site and a 3′ NotI site. The resulting PCR product was digested with NheI and NotI and cloned into the NheI/NotI-digested pCMV6-AC-3HA (Origene Technologies, Inc., Rockville, MD, USA; Cat #PS100010). The subviral vector pNLA1 encoding the NL43 Env WT is a derivative of pNL43, lacking the *gag* and *pol* genes but expressing all other viral genes [20]. For the construction of pNLA1-AD8 WT, encoding the entire AD8 *env* gene in the backbone of pNLA1, we first subcloned the AD8 region between the unique EcoRI and BamHI sites into pUC19. The AD8 Env region was then PCR-amplified from this construct using the 5′ primer 5′-AAGCCATAAT AAGAATTCTG CAACAACTGC TG-3′ (the EcoRI site is underlined) and 3′ primer 5′-AATTCTCCAG GTCTCGAGAT GCTGCTCCCA CCCC-3′ (creating an XhoI site (underlined)). The amplified fragment was cloned into the unique EcoRI and XhoI sites of pNLA1. For the construction of chimera Env (C1), a KpnI/BsmI fragment in pNLA1 was replaced by the corresponding sequence from pAD8. Thus, pNLA1 Env (C1) expressed a chimera where amino acids 43–592 of the 854 amino acid protein were from the R5-tropic AD8 isolate. Chimera Env (C2) was created in a similar manner by inserting a KpnI/BsmI fragment from pNL43 into pNLA1-AD8. Infectious molecular clones pNL43 [21], pAD8 [22], pROD10 [23], and p49.5 (a gift from Bruce Chesebro; NIH Reagent Program Cat# 11389) have been reported previously. The SIVcpz molecular clone pSIVcpzMB897 (Genbank: EF535994) was a gift from Beatrice Hahn [24]. The *env*-defective pNLenv1 (also referred to as NL43DEnv) carries a 1264 bp out-of-frame deletion in the *env* gene that was created on a subclone via digestion with KpnI and BglII and relegation of the blunt-ended sites. The truncated sequence was cloned back into the pNL43 backbone using the unique EcoR1 and BamHI restriction sites. An env-deficient variant of pAD8 (pAD8DEnv) was created by introducing a stop codon immediately downstream of the *vpu* gene via PCR-based mutagenesis using the wild-type constructs as templates and the following mutagenesis primers Env: 5′-ATGATCTGTA GTGCTGCAGA ATAATTGTGG GTCACAGTTT-3′ and 5′-AAACTGTGAC CCACAATTAT TCTGCAGCAC TACAGATCAT-3′. Clones were selected for the gain of an additional PstI site (underlined), and sequences were confirmed using a sequence analysis.

### 2.3. Transmitted/Founder (T/F) Viruses

A panel of ten T/F viruses was obtained from John Kappes through the NIH Reagent Program (CAT# HRP-11919). DNAs were transformed into *E. coli* Stbl2 cells (Thermo Fisher Scientific, Atlanta, GA, USA; Cat# 10268019). We were able to recover eight of the ten viruses (referred to here as TF#1–TF#8). The plasmid DNA of the eight recovered T/F clones was amplified and purified using Qiagen Plasmid Maxi Kits (Qiagen Inc., Germantown, MD, USA; Cat#12263). The purified DNA was used to transfect HEK293T cells for the production of virus stocks.

### 2.4. Antibodies

Human MRC1 (hMRC1) was identified using a rabbit monoclonal antibody to hMRC1 (Abcam Inc., Cambridge, MA, USA, Cat# ab125028). HIV-1 Gag was identified using pooled HIV Ig (NIH Reagent Program; Cat# 3957), cyclophilin A (CypA) was identified using a rabbit polyclonal antibody to CypA (Enzo Life Sciences, Inc., Farmingdale, NY, USA, Cat# BML-SA296), tubulin was identified using a mouse monoclonal antibody to alpha-tubulin (Millipore Sigma, St. Louis, MO, USA; Cat# T9026), and the HA epitope tag was identified using a rat monoclonal antibody to the HA peptide (Millipore Sigma, St. Louis, MO, USA; Cat# 11867431001). HIV-1 Env was detected using an in-house rabbit polyclonal antibody against purified recombinant gp120 from CHO cells.

### 2.5. Transient Transfection of HEK293T Cells

For the transient transfection of HEK293T cells, 3 × 10^6^ cells were plated in a 25 cm^2^ flask and grown overnight. The following day, the cells were transfected using Lipofectamine Plus (Invitrogen; Thermo Fisher Scientific Inc., Waltham, MA, USA) according to the manufacturer’s instructions. The total amounts of plasmid DNAs in all samples were adjusted to 5 µg or 6 µg total DNA as indicated in the text using empty vector DNA where appropriate. After 24 h, the cells were scraped, washed with PBS, suspended in PBS (100 µL/10^6^ cells), and mixed with an equal volume of 2× sample buffer.

### 2.6. Quantitation of Extracellular Virus Using RT Assay

Virus-containing culture supernatants were harvested and precleared via low-speed centrifugation (5 min, 1500 rpm). The supernatants were then filtered through a 0.45 µm syringe filter. Extracellular virus was quantified by measuring the amounts of virus-associated reverse transcriptase (RT) using a ^32^P-based assay as described [25]. We mixed 10 µL of virus-containing culture supernatant with 50 µL of an RT reaction cocktail, which contained, as the template, poly(A) (5 µg/mL), and, as the primer, oligo(dT) [oligo(dT)_12–18_; 0.16 µg/mL] in 50 mM Tris, pH 7.8, 7.5 mM KCl, 2 mM dithiothreitol, 5 mM MgCl_2_, 0.05% NP-40, and a 1 µCi/mL cocktail of [^32^P]dTTP (800 Ci/mmol). Following 90 min incubation at 37 °C, 10 µL of the reaction mixture was spotted onto DEAE ion-exchange paper (Whatman) and washed three times in 2× SSC (0.3 M NaCl and 0.03 M sodium citrate) to remove unincorporated [^32^P]dTTP. Spots were counted in a scintillation counter.

### 2.7. Immunoblot Analysis

For the preparation of cell extracts, cells were washed once with PBS, suspended in PBS (100 µL/10^6^ cells), and mixed with an equal volume of 2× sample buffer (4% SDS, 125 mM Tris pH 6.8, 10% 2-mercaptoethanol, 10% glycerol, 0.001% bromophenol blue). Samples were heated at 95 °C with occasional vortexing until they completely dissolved.

For the preparation of viral extracts, virus-containing supernatants were pelleted via ultracentrifugation, and the viral pellet was suspended directly in 2× sample buffer and heated. Samples were subjected to SDS-PAGE and transferred to polyvinylidene difluoride (PVDF) membranes. The membranes were incubated for 30 min with non-fat dry milk (5% solution in 1× TNT buffer (10 mM Tris pH 7.4, 150 mM NaCl, 0.3% Tween-20)). The membranes were washed once with TNT-N buffer (TNT + 0.05% IGEPAL CA-630), followed by one wash in TNT buffer (2 min each). The membranes were then reacted for 1 h with primary antibodies as described in the text. Unbound antibodies were removed by washing the membranes in TNT-N and TNT (1 wash each, 5 min). The membranes were then incubated with horseradish peroxidase-conjugated secondary antibodies (GE Healthcare, Piscataway, NJ, USA), and proteins were visualized using a Clarity^TM^ Western ECL substrate (Bio-Rad Laboratories, Hercules, CA, USA; Cat# 170-5061) or immobilon Western chemiluminescent HRP substrate (Millipore Sigma, St. Louis, MO, USA; Cat# WBKLS0500). Images were acquired using a ChemiDoc^TM^ imaging system (Bio-Rad Laboratories, Hercules, CA, USA) and were quantified using Image Lab (v 6.0) software.

### 2.8. Viral Infectivity Assay

Virus-containing supernatants were precleared via low-speed centrifugation (5 min, 1500 rpm) and filtered through a 0.45 µm syringe filter. The amounts of virus in the supernatants were quantified using an RT assay. TZM-bl indicator cells (CD4^+^, CCR5^+^, CXCR4^+^) were plated in a 24-well plate (1 mL; 5 × 10^4^ cells/well) and infected with 50 to 150 µL of viral supernatant. Typically, infections were performed in triplicate. After 48 h, the culture medium was aspirated, and cells were lysed in the wells with 200 µL of lysis buffer (25 mM Tris-HCl pH 7.8, 8.0 mM MgCl_2_, 1.0 mM DTT, 1.0% TritonX-100, 15% glycerol). Luciferase activity was determined by combining 10 µL of each lysate with 30 µL of luciferase substrate (Steady-Glo; Promega Corp., Madison, WI, USA). The light emission was measured using a GloMax microplate reader (Promega). The results were corrected for differences in the input virus.

### 2.9. Determination of Viral Tropism

The determination of viral tropism was conducted similar to the viral infectivity assay, except that the assay was performed in 96-well plates, and viruses were pretreated for 2 h at 37 °C with the CXCR4 inhibitor AMD-3100 (1 µM; NIH Reagent Program; Cat#ARP-8128) or the CCR5 inhibitor TAK-779 (1 µM; Cat# ARP-4983) prior to the infection of TZM-bl cells. Luciferase activity was determined 48 h after infection as described for the viral infectivity assay.

### 2.10. OptiPrep Density Gradient Ultracentrifugation

Culture supernatants from transfected HEK293T cells (11 mL) were pelleted in a Beckman ultracentrifuge using an SW41 rotor for 75 min at 35,000 rpm, 4 °C. The pellets were suspended in 500 µL of OPTI-MEM medium (Gibco/Thermo Fisher Scientific, Waltham, MA, USA) and subjected to OptiPrep density gradient centrifugation, essentially as described [26]. Specifically, 9 OptiPrep dilutions (30% to 6% in 3% increments) were prepared by diluting the 60% *w*/*v* stock solution (Millipore Sigma, St. Louis, MO, USA; Cat # D1556) in PBS. Starting with the 30% solution, 445 µL of each dilution was sequentially added to an SW55 tube (total volume = 4005 µL). The gradient was then overlaid with 500 µL of the concentrated culture supernatants and subjected to ultracentrifugation (Beckman; SW55Ti rotor; 45,000 rpm, 90 min at 4 °C). Twelve fractions (380 µL each) were then collected from the top of the gradient and mixed with 150 µL each of 4× sample buffer (8% SDS, 250 mM Tris pH 6.8, 20% 2-mercaptoethanol, 20% glycerol, 0.002% bromophenol blue). The samples were heated to 95 °C for 10 min with occasional vortexing. The samples (120 µL of each fraction) were then run on SDS-PAGE and processed for an immunoblot analysis as described in the text.

### 2.11. Co-Immunoprecipitation Analysis of Env and hMRC1

Cells were harvested 24 h post-transfection and washed twice with cold PBS. The cells were lysed in 1 mL of CoIP lysis buffer (25 mM Tris-HCl (pH 7.4), 150 mM NaCl, 1% NP-40 and 5% glycerol) for 30 min on ice. Insoluble material was removed via centrifugation (5 min, 1500 rpm, 4 °C), and supernatants were collected for subsequent immunoprecipitation. For a co-immunoprecipitation analysis of viral preparations, virus-containing supernatants were pelleted via ultracentrifugation and suspended in CoIP lysis buffer. Ten percent of each lysate was removed and used as an input control; the remaining lysate was used for immunoprecipitation. Precleared cell lysates were incubated with an anti-Env antibody on a rotating wheel overnight at 4 °C and then precipitated with pre-washed GammaBind^TM^ Plus Sepharose^TM^ (Millipore Sigma, St. Louis, MO, USA; Cat# GE17-0886-02). Samples were washed three times with CoIP lysis buffer. Proteins were eluted by heating beads in 1x sample buffer for 10 min at 95 °C with occasional vortexing. Eluted samples were subjected to SDS-PAGE and an immunoblot analysis.

### 2.12. Glycoprotein Analysis

For a glycoprotein analysis of hMRC1, exogenously expressed untagged hMRC1 was purified from transfected HEK293T cells as follows: Cells were washed once with PBS and lysed in 500 µL of TritonX-100-based lysis buffer. The samples were incubated in lysis buffer for 20 min at 4 °C on a rotator. Insoluble material was then removed via centrifugation at 15,000 rpm for 10 min. Ten percent of the clarified supernatant was used as an input control. The remaining lysate was incubated for 2 h at 4 °C on a rotator with Concanavalin A (ConA) agarose (Vector Laboratories, Burlingame, CA, USA; Cat# AL-1003). ConA-hMRC1 complexes were washed three times with TritonX-100 lysis buffer. Half the samples were left untreated, while the second half was treated with endoglycosidase H (EndoH). For an EndoH analysis, digestion was performed directly on hMRC1 bound to ConA agarose. All reagents and buffers were provided by the manufacturer (New Engaland Bio Labs, Ipswich, MA, USA; Cat# P0702). Samples were first washed once with denaturing buffer, then suspended in denaturing buffer, and heated at 95 °C for 10 min. Glyco buffer was then added along with an excess of enzyme (2500 units of Endo H), and samples were incubated at 37 °C for 1 h. Bound proteins were eluted from beads by boiling in an equal volume of sample buffer for 10 min at 95 °C and analyzed via immunoblotting.

## 3. Results

### 3.1. Exogenous Expression of hMRC1 Attenuates Virus Infectivity in an Isolate-Specific Manner

**Figure 1:**
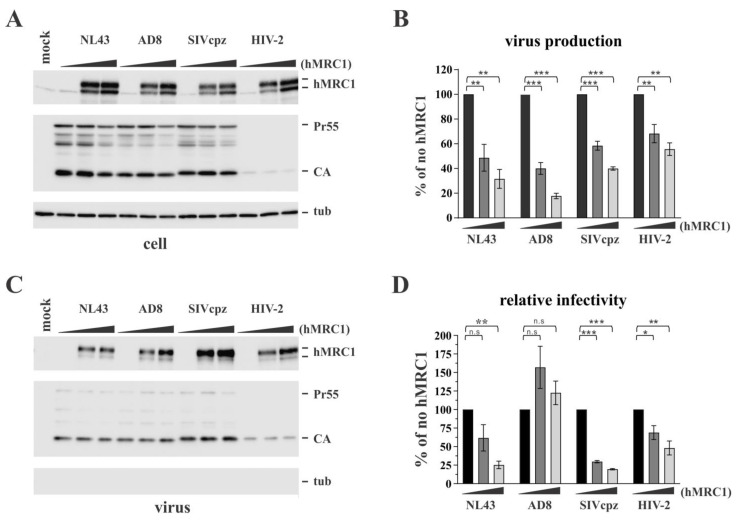
hMRC1 broadly inhibits lentivirus production but attenuates viral infectivity in a virus isolate-specific manner. HEK293T cells were transfected with 3.0 µg of pNL43, pAD8, pSIVcpzMB897 (SIVcpz), or pROD10 (HIV-2) together with increasing amounts of pCMV6-hMRC1 (0 [solid black bars], 0.5 [dark grey bars], 2.0 µg [light grey bars]). Total amounts of transfected plasmid DNAs were adjusted to 5.0 µg for each sample using empty vector DNA as needed. Cells and virus-containing supernatants were harvested 24 h post-transfection. (**A**) Cell extracts were subjected to immunoblot analysis using antibodies to hMRC1, pooled HIV-1 patient serum (Pr55, CA), or tubulin. (**B**) Virus production was measured as reported previously [18] by quantifying virus-associated reverse transcriptase (RT) activity using clarified and filtered culture supernatants. Virus production in the absence of hMRC1 was defined as 100% for each virus (solid black columns). The results are shown as an average of three independent experiments with SEM. (**C**,**D**) To determine viral infectivity, filtered culture supernatants were used for the infection of TZM-bl indicator cells. Input virus was adjusted to equal RT activity and validated using immunoblot analysis. The absence of cellular contaminants was verified by the absence of tubulin in the samples (**C**). (**D**) Luciferase activity was measured 48 h later, and values were normalized for input virus. Luciferase activity observed in the absence of hMRC1 (no hMRC1) was defined as 100% for each virus (solid black columns). The results are shown as an average of three independent experiments with SEM. Statistical significance in panels (**B**,**D**) was determined using one-way ANOVA with Dunnett’s post hoc test. The null hypothesis was rejected if the *p* value was less than the significance levels, i.e., * α ≤ 0.05; ** α ≤ 0.01; *** α ≤ 0.001; n.s: non-significant.

We previously identified human mannose receptor 1 (hMRC1) as an antiviral factor that broadly inhibits lentiviral particle release in a BST-2-like manner [18]. Surprisingly, we found at the time that the effect of hMRC1 on particle release did not require the expression of the Env glycoprotein. However, since hMRC1 is capable of binding to HIV-1 Env, we wanted to investigate the potential effects of hMRC1 on Env function. We first analyzed the effect of increasing amounts of hMRC1 on the production and infectivity of four different lentiviruses, namely, HIV-1_NL43_ (NL43), HIV-1_AD8_ (AD8), SIVcpz_MB897_ (SIVcpz), and HIV-2_ROD10_ (HIV-2), using transiently transfected HEK293T cells, which do not express endogenous hMRC1. NL43 and HIV-2 are X4-tropic, while AD8 and SIVcpz are R5-tropic [27,28]. We confirmed the tropism of these viruses experimentally by measuring their sensitivity or insensitivity to the CCR5 inhibitor TAK-779 or the CXCR4 inhibitor AMD-3100 (Supplemental Figure S1A). We employed immunoblot analyses to gauge the expression of hMRC1 and to assess the synthesis and release of viral Gag proteins (Figure 1A,B). Consistent with our previous observation [18], the transient overexpression of hMRC1 did not significantly affect the synthesis of viral proteins (Figure 1A, CA and Pr55), even though HIV-2 Gag was only inefficiently recognized by the pooled HIV-1 serum. In contrast, exogenously expressed hMRC1 inhibited the release of all four lentiviruses, as measured via reverse transcriptase (RT) activity, in a dose-dependent manner (Figure 1B). Of note, hMRC1 was readily detected in both the cell extracts (Figure 1A) and the concentrated virus samples (Figure 1C). To assess the effect of hMRC1 on the infectivity of these viruses, input samples were normalized based on RT activity, validated using immunoblotting (Figure 1C), and used to infect TZM-bl indicator cells (Figure 1D). We found that the relative infectivity, after the normalization of the input virus, of HIV-1_NL43_, SIVcpz_MB897_, and HIV-2_ROD10_ decreased with increasing levels of hMRC1. In contrast, the infectivity of HIV-1_AD8_ did not change in a statistically significant manner (Figure 1D). These results suggest that hMRC1 has the ability to attenuate viral infectivity in an isolate-specific manner and, thus, through a mechanism that is distinct from the one regulating virus particle release.

### 3.2. R5-Tropic T/F Viruses Vary in Their Sensitivity to Inhibition by hMRC1

**Figure 2:**
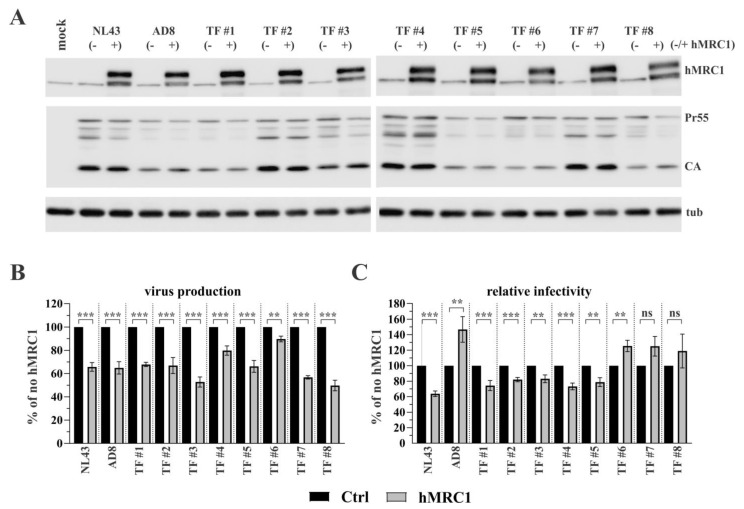
Sensitivity of T/F viruses to inhibition by hMRC1. Eight T/F viruses (TF#1–TF#8) were obtained from John Kappes through the NIH Reagent Program as described in the Section 2. Plasmid DNA (3 µg) from each of the eight T/F viruses was transfected into HEK293T cells either in the presence of 2 µg of empty vector (-) or pCMV6-hMRC1 (+). Cells and virus-containing supernatants were collected 24 h later. (**A**) Cell extracts were subjected to immunoblot analysis using antibodies to hMRC1, pooled HIV-1 patient serum (Pr55, CA), or tubulin. (**B**) Virus production was quantified as in Figure 1B. Virus production in the absence of hMRC1 was defined as 100% for each virus (solid black columns). The results are shown as an average of three independent experiments with SEM. (**C**) Inhibition of viral infectivity by hMRC1 was measured as in Figure 1D. Luciferase activity observed in the absence of hMRC1 (no hMRC1) was defined as 100% for each virus (solid black columns). The results are shown as an average of three independent experiments with SEM. Statistical significance in panels (**B**,**C**) was determined using a Student’s *t*-test. ** α ≤ 0.01; *** α ≤ 0.001; n.s: non-significant.

In addition to the four highly lab-adapted viruses analyzed above, we tested a panel of eight T/F viruses, which were reported to efficiently replicate in primary CD4+ T lymphocytes and, albeit less efficiently, in macrophages using the CCR5 coreceptor [29]. We experimentally confirmed the R5 tropism of the T/F viruses via their sensitivity to the CCR5 inhibitor TAK-779 and insensitivity to the CXCR4 inhibitor AMD-3100 (Supplemental Figure S1B). As with the lab-adapted viruses, the expression of hMRC1 had no significant effect on viral Gag protein synthesis (Figure 2A; CA and Pr55), although there were differences among T/F isolates with regard to overall Gag expression. As with the lab-adapted viruses, the expression of hMRC1 inhibited the release of viruses from transfected 293T cells (Figure 2B). Interestingly, hMRC1 expression inhibited the infectivity of T/F viruses TF#1-TF#5 when normalized for the input virus, while TF#6-TF#8 viruses were not inhibited by hMRC1 (Figure 2C). Indeed, as with the AD8 virus, the infectivity of TF#6, TF#7, and TF#8 was slightly increased, although the effect did not reach statistical significance for TF#7 and TF#8. Together with the results in Figure 1, these results confirm that the inhibition of viral infectivity by hMRC1 is virus isolate-specific.

### 3.3. The Env V3 Region Is One of the Key Determinants for hMRC1-Mediated Attenuation of Virus Infectivity

**Figure 3:**
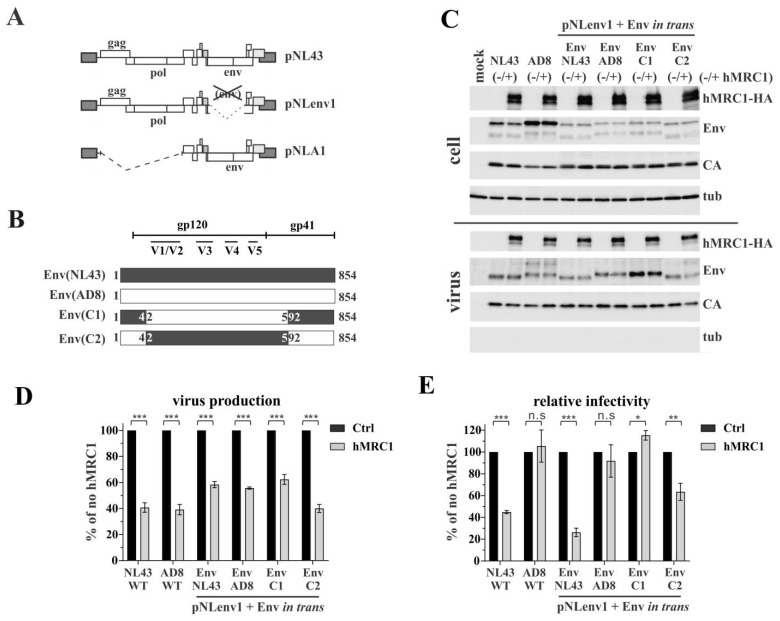
The central variable region of NL43 Env is important for the hMRC1-mediated attenuation of viral infectivity. (**A**) Schematic of vectors involved in this analysis. The full-length infectious molecular clone, pNL43, is shown for reference. Plasmid pNLenv1 is an *env*-defective pNL43 derivative carrying a 1264 bp out-of-frame deletion in the *env* gene. pNLA1 is a subviral vector expressing all viral proteins, except for Gag and Pol [31]. This vector was used for construction of the Env chimera shown in panel (**B**). (**B**) Schematic presentation of the envelope chimera constructed from NL43 and AD8 env in the backbone of pNLA1. The black and white boxes indicate NL43 *env* and AD8 *env* regions, respectively. Numbers indicate amino acid positions of the fusions. Variable regions in Env are indicated on the top. (**C**–**E**) For the analysis of chimeric Env constructs, HEK293T cells were transfected with 3 µg of Env-defective pNLenv1 and 2 µg of pNLA1 (Env(NL43)), pNLA1-AD8 (Env(AD8)), pNLA1-NL-AD8-NL (Env(C1)), or pNLA1-AD8-NL-AD8 (Env(C2)) in the absence or presence of 1 µg of pCMV6-hMRC1-HA. Full-length pNL43 or pAD8 (3 µg) with or without of 1 µg of pCMV6-hMRC1-HA was included as control. Total amounts of transfected plasmid DNA were adjusted to 6 µg for each sample using empty vector DNA as needed. Cells and virus-containing supernatants were harvested 24 h post-transfection and used to control protein expression (**C**), or determine virus production (**D**) or viral infectivity (**E**) as described in Figure 1 caption. Virus production and viral infectivity in the absence of hMRC1 was defined as 100% for each virus (solid black columns). The results are shown as an average of three independent experiments with SEM. Statistical significance in panels (**B**,**C**) was determined using a two-tailed, paired Student’s *t* test. The null hypothesis was rejected if the *p* value was less than the significance levels, i.e., * α ≤ 0.05; ** α ≤ 0.01; *** α ≤ 0.001; n.s: non-significant.

To gain a more detailed understanding of how hMRC1 can modulate viral infectivity, we constructed chimeric Env expression vectors by exchanging portions of the *env* genes (corresponding to amino acid residues 42 to 592) of the X4-tropic HIV-1_NL43_ Env and the R5-tropic HIV-1_AD8_ Env in the backbone of the subviral vector pNLA1 (Figure 3A) as schematically shown in Figure 3B. The immunoblot analysis of cell extracts revealed no effect of hMRC1 on intracellular Gag or Env expression and, consistent with the data shown in Figure 1, revealed the presence of hMRC1 in viral supernatants (Figure 3C). Of note, the Env protein of AD8 exhibits a somewhat slower mobility relative to that of the NL43 or 49.5 Envs, possibly due to differential glycosylation. The slower mobility of Env was also apparent in chimera C1, which encodes residues 42 to 592 of the AD8 Env.

The sensitivity of viruses encoding the chimeric Envs to hMRC1-mediated attenuation was then tested. HEK293T cells were transfected with an Env-defective pNL43 variant, pNLenv1, together with the pNLA1-based Env expression vectors depicted in Figure 3B, in the presence or absence of hMRC1 (Figure 3D,E). The full-length molecular clones HIV-1_NL43_ and HIV-1_AD8_ served as positive and negative controls, respectively. Consistent with the results in Figure 1, hMRC1 expression inhibited virus release, even when Env was expressed *in trans* (Figure 3D). Also, as before, hMRC1 inhibited the infectivity of full-length HIV-1_NL43_ but not HIV-1_AD8_ (Figure 3E). Again, this was true even when the NL43 and AD8 Env proteins were expressed *in trans* together with the Env-defective pNLenv1 vector. Of note, chimera C1, carrying the central region of AD8 Env, was insensitive to hMRC1. Conversely, chimera C2, encoding the central region of NL43 Env, remained hMRC1-sensitive. These results further confirm the isolate-specific effect of hMRC1 on viral infectivity and suggest that the determinants of hMRC1 sensitivity in Env map to the variable region of the Env ectodomain. The above noted differences in electrophoretic mobility, however, do not account for their differential sensitivities to hMRC1.

**Figure 4:**
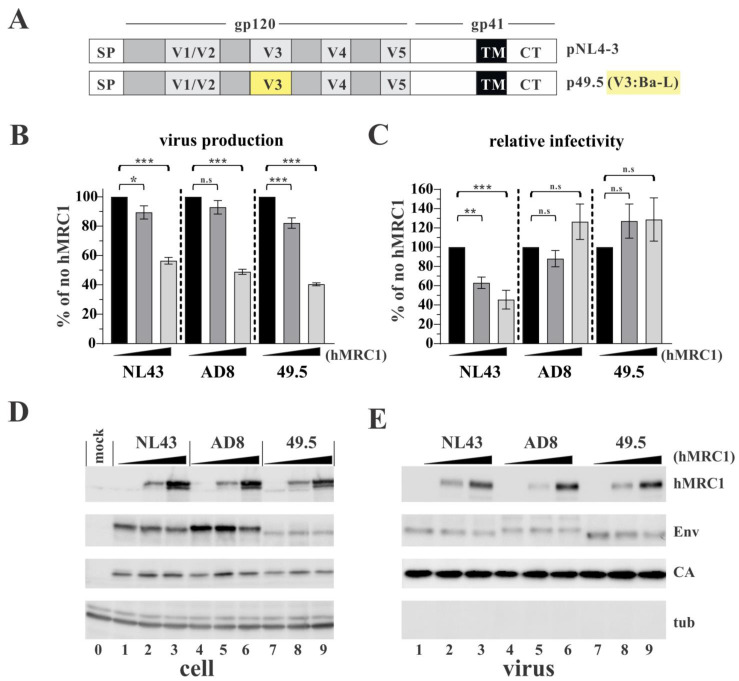
The Env V3 region is a key determinant for hMRC1-mediated attenuation of viral infectivity. (**A**) Schematic presentation of the Env region carrying the V3 region from the R5-tropic Ba-L isolate in the backbone of the X4-tropic NL43 clone. The Ba-L region is highlighted in yellow. (**B**,**C**) HEK293T cells were transfected with 4 µg of pNL43 (X4-tropic), pAD8 (R5-tropic), or p49.5 (R5-tropic) viral vectors together with increasing amounts of pCMV6-hMRC1 (0 [solid black bars], 0.1 [dark grey bars], 1.0 µg [light grey bars]). Total amounts of transfected plasmid DNA were adjusted to 5 µg for each sample using empty vector DNA as needed. Cells and virus-containing supernatants were harvested 24 h post-transfection and used to determine virus production (**B**) or viral infectivity (**C**) as described in Figure 1 caption. Virus production and viral infectivity in the absence of hMRC1 was defined as 100% for each virus (solid black columns). The results are shown as an average of three independent experiments with SEM. Statistical significance in panels (**B**,**C**) was determined using one-way ANOVA with Dunnett’s post hoc test. The null hypothesis was rejected if the *p* value was less than the significance levels, i.e., * α ≤ 0.05; ** α ≤ 0.01; *** α ≤ 0.001; n.s: non-significant. (**D**,**E**) Whole-cell extracts and extracts of concentrated viral supernatants were prepared 24 h post-transfection, and samples were processed for immunoblotting using antibodies to hMRC1, Envelope (Env), and HIV-1 Gag (CA). A tubulin blot (tub) was included as a loading control. Note that samples shown in panel (**E**) and used for the infectivity assay (panel (**C**)) were adjusted to account for the difference in virus release observed in panel (**B**). A representative result from three independent experiments is shown.

To further narrow down the region in Env responsible for sensitivity to hMRC1, we tested an HIV-1 hybrid clone, p49.5 [30], with R5 coreceptor usage. The 49.5 variant carries just the V3 region of the R5-tropic Ba-L isolate in the backbone of pNL43 (Figure 4A, yellow highlight). Predictably, hMRC1 inhibited the secretion of all viruses (Figure 4B), and, as expected, the infectivity of the NL43 virus decreased with increasing levels of hMRC1 (Figure 4C). Importantly, however, hMRC1 did not inhibit the infectivity of AD8 and 49.5 viruses. If anything, hMRC1 slightly stimulated the infectivity of AD8 and 49.5 viruses, although the effect was statistically not significant (Figure 4C). The expression of intracellular gag proteins was not affected by increasing amounts of hMRC1 (Figure 4D, CA). Moreover, the levels of cell-associated and virus-associated Env protein were not affected by increasing hMRC1 levels. However, the absolute levels of Env observed for the three virus isolates differed, possibly due to differences in the affinity of the ENV-specific antibody used in the experiment. Together, these results confirm that the effect of hMRC1 on viral infectivity is virus isolate-specific and maps to the V3 region in Env. These results also indicate that the effect of hMRC1 on viral infectivity is not caused by reduced Env expression, altered Env maturation, or Env packaging into virus particles.

### 3.4. hMRC1 Interacts with NL43 Env but Not Env from AD8 and 49.5

**Figure 5:**
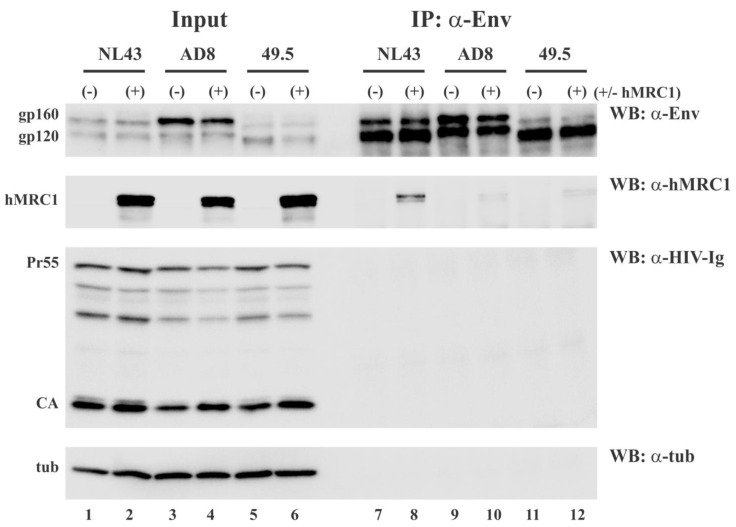
The hMRC1-mediated attenuation of viral infectivity correlates with an increased binding affinity of hMRC1 to HIV-1 Env. HEK293T cells were transfected with 4.0 µg of pNL43, pAD8, or p49.5 together with (+) or without (-) of 1.0 µg of pCMV6-hMRC1. Total amounts of transfected plasmid DNA were adjusted to 5.0 µg for each sample using empty vector as needed. Cells were harvested 24 h post-transfection and processed for immunoprecipitation analysis. Whole-cell extracts (input) and samples immunoprecipitated with antibodies to HIV-1 Env (IP:a-Env) were subjected to immunoblot analysis using antibodies to Env, hMRC1, HIV-1 Gag, and tubulin. A representative result from three independent experiments is shown.

The apparent role of the V3 region in the isolate-specific inhibition of viral infectivity could suggest a mechanism by which hMRC1 interferes with the normal function of the Env protein through the binding to the V3 region in Env, thereby competing for the binding to chemokine receptors on the target cell. To test this possibility, we performed a co-immunoprecipitation analysis of Env and hMRC1. HEK293T cells were transfected with full-length molecular clones NL43, AD8, or 49.5 either in the presence (+) or absence (-) of hMRC1. The transfected cells were collected 24 h later and processed for immunoprecipitation with an Env-specific antibody as described in the Section 2. A fraction of the cell lysates (10%) was set aside prior to immunoprecipitation as an input control. The input control samples and immunoprecipitates were subjected to SDS-PAGE, transferred to PVDF membranes, and probed by immunoblotting (WB) with antibodies to Env, hMRC1, tubulin (tub), or HIV-1 Ig (Pr55; CA) (Figure 5). The Env proteins from all three virus isolates were efficiently precipitated by the Env-specific antibodies (Figure 5, lanes 7–12). As expected, neither cellular tubulin nor HIV-1 Gag proteins interacted with HIV-1 Env in this pulldown assay. Interestingly, however, NL43 Env was able to pull down hMRC1, while interaction with AD8 or 49.5 Env was near the background. These results support our hypothesis that the virus isolate-specific inhibition of viral infectivity by hMRC1 involves an interaction of Env and hMRC1 and, thus, could suggest a mechanism of competitive interference.

### 3.5. hMRC1 Is Packaged into Viral Particles in an Env Independent Manner

**Figure 6:**
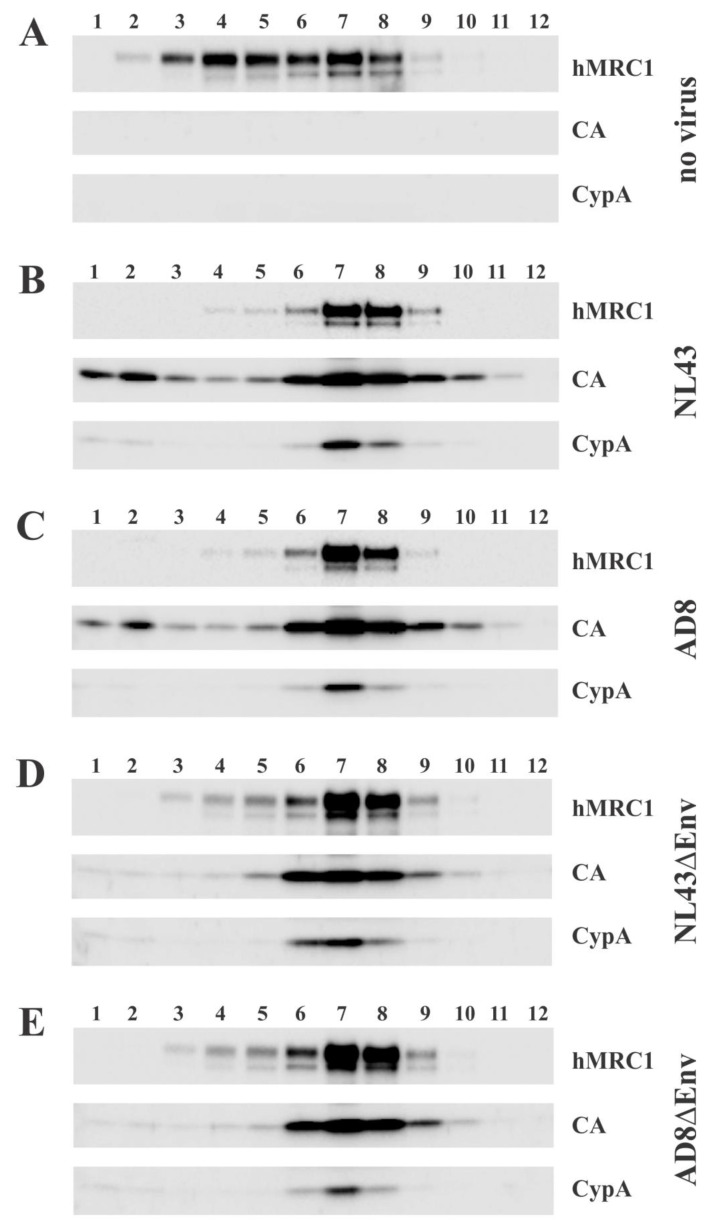
Exogenously expressed hMRC1 is incorporated into viral particles with or without Env. OptiPrep density gradient analysis of extracellular hMRC1 in the absence or presence of virus particles. HEK293T cells were transfected with 1.0 µg of pCMV6-hMRC1 together with 4.0 µg of empty vector (**A**), pNL43 (**B**), pAD8 (**C**), pNLenv1 (pNL43DEnv) (**D**), or pAD8Denv (**E**). Supernatants from each culture were collected 24 h post-transfection and processed for OptiPrep density gradient analysis as detailed in the Section 2. Twelve fractions were collected and subjected to SDS-PAGE and immunoblot using antibodies to hMRC1 and HIV-1 Gag (CA). A cyclophilin A (CypA) blot was used as a positive control for a virus particle-associated host protein. Shown is a representative experiment from three independent analyses.

The observation that hMRC1, when expressed in the donor cell, can inhibit the infectivity of viruses carrying an X4-tropic envelope without obvious effects on the expression levels of viral Env raises the question of whether this phenomenon is associated with the incorporation of hMRC1 into nascent virions. Our immunoblot of pelleted virions in Figure 1C, Figure 3C and Figure 4E is consistent with the presence of hMRC1 in cell-free virions. To further test this possibility, we prepared concentrated cell-free viruses from the supernatants of transiently transfected HEK293T cells and subjected them to OptiPrep gradient centrifugation as described in the Section 2.10 (Figure 6). Endogenously expressed cyclophilin A (CypA) was used as a control since it is known to be incorporated into viral particles [32]. Our analysis included wildtype NL43 (panel B) and wildtype AD8 viruses (panel C); in addition, we analyzed Env-defective NL43 (NL43DEnv; panel D) and Env-defective AD8 (AD8DEnv; panel E). Finally, we included as a control concentrated supernatant from HEK293T cells transfected with just the hMRC1 vector (i.e., no virus; panel A). We found that, in the absence of virus production (panel A), hMRC1 was secreted into culture supernatants in a pelletable form and broadly partitioned in the OptiPrep gradient between fractions 2 and 8 without an obvious concentration anywhere in the gradient. As expected, CypA was not observed in the absence of virus production. The OptiPrep gradient analysis of hMRC1 in the presence of virus production resulted in a clear concentration of hMRC1 in fractions containing viruses or CypA (panels B-E; fractions 6–8). The co-partitioning of hMRC1 with virus-containing fractions was virus isolate-independent and not affected by the presence or absence of Env. From these data, we conclude that hMRC1 is packaged into viral particles in an Env-independent manner.

### 3.6. Virus-Incorporated hMRC1 Binds to NL43 Env but Only Weakly Interacts with the Env Proteins of AD8 and the Closely Related 49.5 Virus

**Figure 7:**
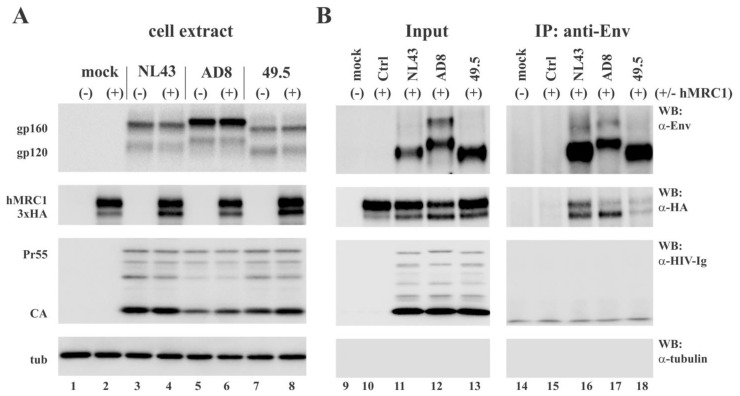
Virus-incorporated hMRC1 specifically interacts with NL43 Env. HEK293T cells were transfected with 4.0 µg of pNL43, pAD8, or p49.5 together with (+) or without (-) of 2.0 µg of pCMV6-AC-hMRC1-3xHA. Total amounts of transfected plasmid DNA were adjusted to 6.0 µg for each sample using empty vector as needed. Cells were harvested 24 h post-transfection and processed for immunoprecipitation analysis as described in Figure 5 caption. (**A**) Whole-cell extracts were subjected to SDS-PAGE and processed for immunoblot analysis as described in the Section 2.7. Membranes were probed with antibodies to Env, HA (hMRC1), tubulin, or with HIV-Ig. Proteins are identified on the right. (**B**) Filtered cell-free supernatants were pelleted in a Beckman ultracentrifuge (SW41; 75 min, 35,000 rpm, 4 °C). Pelleted material was suspended in 1 mL of CoIP lysis buffer, and 100 µL was set aside as input control. The remaining lysates were immunoprecipitated with Env-specific antiserum. Input samples and immunoprecipitated samples were subjected to SDS-PAGE and transferred to PVDF membranes. Membranes were then probed with antibodies to Env, HA (hMRC1), tubulin, or HIV-Ig. A representative result from three independent experiments is shown.

One possible mechanism of how hMRC1 might attenuate viral infectivity is through the physical binding of hMRC1 to HIV-1 Env in a manner that would prohibit Env from performing its critical role in the early steps of virus infection. To test whether virion-incorporated hMRC1 interacts with NL43 Env similar to cell-associated Env and hMRC1 shown in Figure 5, we performed co-immunoprecipitation analyses on concentrated supernatants from virus-producing cells to identify the virus-associated complexes of Env and hMRC1 (Figure 7B). To increase the sensitivity of the assay, the current experiment was performed using C-terminally HA-tagged hMRC1 (hMRC1-3xHA) as opposed to untagged hMRC1, which was used in Figure 5. Of note, untagged (e.g., Figure 1, Figure 2 and Figure 4) and HA-tagged hMRC1 (e.g., Figure 3) both inhibit virus release and have the ability to reduce viral infectivity. HEK293T cells were transfected with pNL43, pAD8, or p49.5 molecular clones in the presence or absence of hMRC1-3xHA. Cell extracts were then processed for immunoblotting, which revealed that the expression of hMRC1 was comparable (Figure 7A, lanes 2, 4, 6, and 8) and had no negative impact on the expression of Gag (Pr55; CA) and Env (gp160/gp120) (Figure 7A, compare lanes 3 and 4; 5 and 6; and 7 and 8). Mock or hMRC1-only transfected cells were included as specificity controls (Figure 7A, lanes 1 and 2). Filtered cell-free supernatants were then concentrated via ultracentrifugation and suspended in 1 mL of lysis buffer. An aliquot (10%) was retained as an input control (Figure 7B, lanes 9–13). The remainder of the extracts was immunoprecipitated with an HIV-1 Env-specific antibody (Figure 7B, lanes 14–18), and precipitates were subjected to immunoblotting using antibodies to Env, HA, HIV-Ig, or alpha tubulin. HIV-1 Env from all three virus isolates was efficiently immunoprecipitated by the Env-specific antibody (Figure 7, top panel, lanes 16–18). As expected, HIV-1 Gag proteins as well as tubulin did not co-immunoprecipitate with the Env-specific antibody (Figure 7B, lower two panels, lanes 14–18). Importantly, hMRC1 co-immunoprecipitated with NL43 Env (Figure 7B, second panel, lane 16) but only weakly interacted with the Env proteins of the R5-tropic AD8 and 49.5 isolates (Figure 7B, second panel, lanes 17–18). Of note, we found that the transiently expressed HA-tagged hMRC1 produces a double band. The endoglycosidase H (EndoH) analysis indicates that the slower migrating form (upper band) of hMRC1 is EndoH-resistant and, therefore, most likely represents a mature protein carrying complex carbohydrates that has exited the endoplasmic reticulum (ER) [33]. In contrast, the faster migrating form (lower band) is EndoH-sensitive and, thus, most likely reflects immature high-mannose hMRC1 that has not yet exited the ER (Supplemental Figure S2). We conclude that mature hMRC1 interacts selectively with NL43 Env both in cell extracts (Figure 5, lane 8) and in cell-free virions (Figure 7B, lane 16) but does not, or only inefficiently, interacts with R5-tropic AD8 and 49.5 Env (Figure 6B, lanes 17–18).

## 4. Discussion

The current study is an extension of our previous work, where we found that hMRC1 can inhibit the release of newly assembled virions from infected cells, resulting in the accumulation of viral particles on the cell surface [18]. While the mechanistic basis for the retention of viral particles by hMRC1 remains unknown, its phenotype is quite similar to the effect reported for BST-2 [18]. BST-2 tethers viral particles to the surface of virus-producing cells [34,35] and is thought to inhibit the dissemination of HIV-1 into the periphery, thereby facilitating the cell-to-cell spread of the virus in infected tissues (reviewed in [36,37]). The observation that HIV-1 employs multiple mechanisms to downmodulate hMRC1 expression in infected macrophages [16,17,18] suggests that HIV-1 has a vested interest in bypassing the restriction(s) imposed by hMRC1 in infected macrophages.

The premise of the current study was to see whether, aside from inhibiting virus particle release, hMRC1 imposes additional restrictions on virus replication. Since the inhibition of hMRC1 by HIV-1 occurs primarily at the transcriptional level [16,17,18], our approach was to overexpress hMRC1 from a heterologous (CMV) promoter that should be insensitive to viral countermeasures and document its consequences for virus propagation in HEK293T cells, which do not express endogenous hMRC1. Not surprisingly, we found that the overexpression of hMRC1 resulted in a dose-dependent inhibition of virus particle release irrespective of the virus isolate (Figure 1, Figure 2, Figure 3 and Figure 4). Surprisingly, however, when we assessed the infectivity of the viruses produced in the presence of hMRC1, we found that some but not all viruses were inhibited by hMRC1. To better understand this phenomenon, we used NL43 as our hMRC1-sensitive prototype and AD8 and 49.5 isolates as our hMRC1-resistent controls since our Env-specific antibody interacted well with the Env proteins from these isolates (Figure 3, Figure 4, Figure 5 and Figure 7). The SIVcpz and HIV-2 isolates, while clearly restricted by hMRC1 (Figure 1D), were not further characterized since antibodies to their Env proteins were not available.

Using OptiPrep gradients, we confirmed our previous observation that hMRC1 is incorporated into viral particles ([18]; Figure 6 and Figure 7). The mechanism of hMRC1 virion incorporation remains to be investigated. However, we can already conclude that it is not dependent on HIV-1 Env since Env-defective virions contain comparable amounts of hMRC1 to Env-containing virions (Figure 6). Also, as shown in Figure 7B, HIV-1 Env and hMRC1 can both be present in virions, but only the NL43 Env interacts with hMRC1 strong enough to be detectable via co-immunoprecipitation. It seems therefore likely that, like Env itself, the packaging of hMRC1 is relatively non-specific and is a consequence of its mere presence on the surface of virus-producing cells rather than being packaged through interaction with another viral component such as Gag. This does not exclude the possibility of the Env-hMRC1 complex in the NL43 sample forming in the virus-producing cell and is packaged as a pre-existing complex. Unfortunately, we do not currently have the experimental tools to discriminate between the two options.

Interestingly, co-immunoprecipitation studies revealed a preferential interaction of hMRC1 with the hMRC1-sensitive NL43 Env but not the hMRC1-insensitive controls. This interaction was observed both intracellularly (Figure 5) and in cell-free virions (Figure 7B). The interaction of HIV-1 Env with hMRC1 is likely to occur *in cis*. This conclusion is based on our observation that HIV-1 Env and hMRC1 failed to co-immunoprecipitate when the proteins were expressed in separate cells and cell lysates were mixed prior to immunoprecipitation. Since the hMRC1-sensitive NL43 and the insensitive 49.5 viruses only differ in their Env V3 region (Figure 4A), it is likely that the Env/hMRC1 interaction occurs through the V3 domain both intracellularly and in cell-free virions. While the V3 region in Env is important for interaction with chemokine receptors, sensitivity to hMRC1 does not appear to correlate with viral tropism. In particular, all eight T/F viruses are R5-tropic (Supplemental Figure S1B); yet, five of them (TF #1–TF #5) are sensitive to hMRC1, whereas TF #6 to TF #8 are insensitive to hMRC1 (Figure 2C). Nevertheless, since the V3 domain in HIV-1 Env is critical for the engagement of its chemokine coreceptor during virus entry into a target cell, it is tempting to speculate that the binding of hMRC1 to the V3 domain of hMRC1-sensitive Envs in viral particles interferes with this process. The reason why hMRC1 does not bind to all Envs is not clear. However, since hMRC1 contains eight tandem repeats of a carbohydrate recognition domain [3], it seems possible that the differential glycosylation of HIV-1 Env may be responsible. A previous study suggested that a region in Env known as the mannose patch may mediate the interaction between Env and hMRC1 [16]. Such mannose patches were reported for the hMRC1-sensitive NL43 virus as well as the hMRC1-insensitive AD8 virus [16], indicating that they do not account for the species-specific inhibition of viral infectivity by hMRC1. Instead, the binding of hMRC1 to Env may induce steric hindrance for the engagement of the chemokine receptor during infection, or it could cause a conformational change in Env with the same effect on chemokine receptor engagement. The alignment of the V3 domains of the 13 viruses used in this study revealed a significant variation in their amino acid sequences (Supplemental Figure S3). The experimentally determined tropism of our isolates (Supplemental Figure S1) did not align with predictive models, such as the 11/25 rule, which predicts X4 tropism if a positively charged amino acid is observed at position 11 or 25 of the V3 sequence [38]. More importantly, we could not find any sequence motifs in the V3 regions that would correlate with sensitivity to inhibition by hMRC1. It seems therefore more likely that the sensitivity of HIV-1 infectivity to hMRC1 is dictated by secondary or tertiary structural motifs in the Env V3 region. What is the possible physiological relevance of our results? It is true that the lab-adapted X4-tropic NL43 isolate does not normally infect macrophages and, therefore, will not under normal circumstances incorporate hMRC1. However, all eight T/F viruses used in our study were found to utilize the CCR5 coreceptor for entry into primary human CD4+ T lymphocytes and MDM [29]. In addition, some syncytia-inducing HIV-1 variants have the ability to initially use both CCR5 and CXCR4 coreceptors, but the ability to use the CCR5 coreceptor is eventually lost [39]. Thus, T/F viruses and other dual-tropic viruses could indeed enter macrophages and package hMRC1, which would reduce their ability to infect CD4+ T cells and affect replication in macrophages. Preliminary attempts to demonstrate the packaging of hMRC1 into virions from macrophages infected with T/F viruses were inconclusive due to the poor replication efficiency of these viruses in macrophages and the overall low levels of hMRC1. Nevertheless, we can conclude that the HIV-1-induced downmodulation of hMRC1 in infected macrophages would have the dual benefit of enhancing virus release and maximizing the ability of such viruses to infect and replicate in CD4+ T lymphocytes. One limitation of our study is that the effects of hMR1 on viral infectivity were tested exclusively using the TZM-bl indicator cell line. Since the entry of HIV is highly dependent on receptor/coreceptor density, it remains to be tested whether the findings in the current study can be observed in relevant cell types such as primary T cells.

## Data Availability

All reagents are freely available by request from the corresponding author.

## Supplementary Materials

The following supporting information can be downloaded at https://www.mdpi.com/article/10.3390/v15102057/s1, Figure S1: Determination of viral tropism; Figure S2: Endoglycosidase H (EndoH) analysis of transiently expressed hMRC1; Figure S3: Alignment of Env V3 domains of viruses used in this study.
