# Supplementary material for: Human Mannose Receptor 1 Attenuates HIV-1 Infectivity in a Virus Isolate-Specific Manner
Source: Viruses. 2023 Oct 6;15(10):2057. doi: 10.3390/v15102057 (PMC10612104; doi:10.3390/v15102057)
Supplement: Supplementary file 1 [file viruses-15-02057-s001.zip › viruses-2527314-supplementary.pdf]

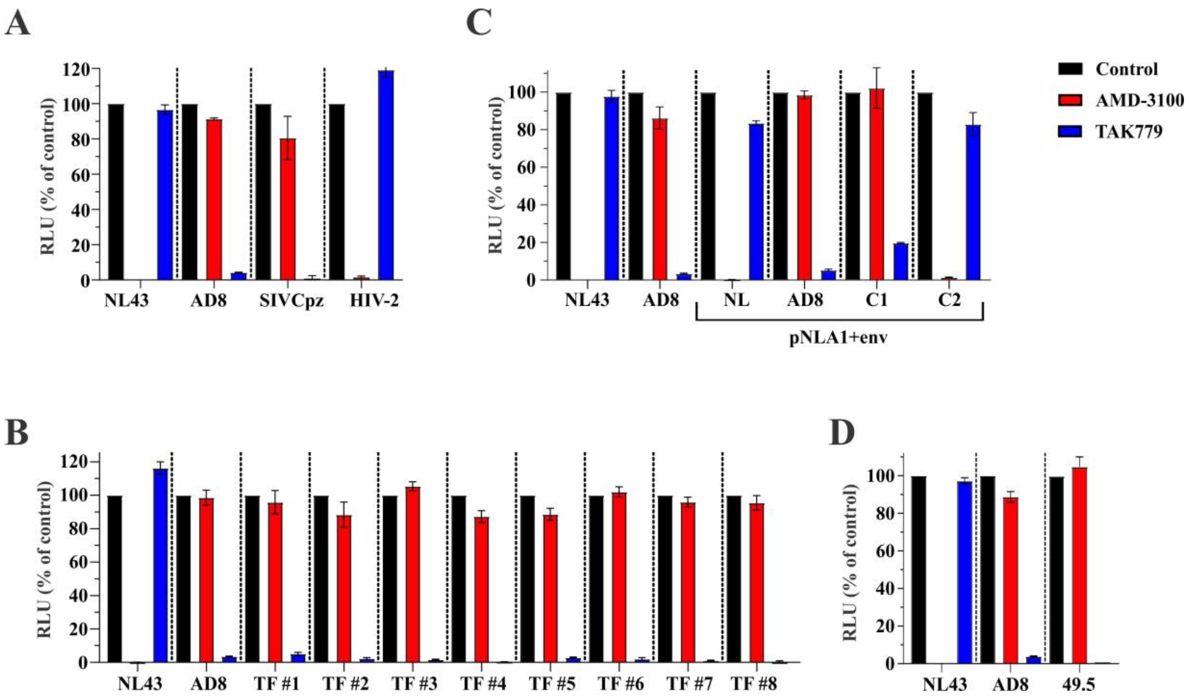

Supplemental Figure S1. Determination of viral tropism. The tropism of the viruses used in this study (figures 1-4) was verified by determining their sensitivity to inhibitors of CXCR4 (AMD-3100) or CCR5 (TAK-779) as described in Materials and Methods. **(A)** Tropism of viruses used in figure 1. **(B)** Tropism of T/F viruses used in figure 2. **(C)** Tropism of viruses used in figure 3. **(D)** Tropism of viruses used in figure 4. Experiments were performed once in triplicate and the results are shown as mean plus SEM.

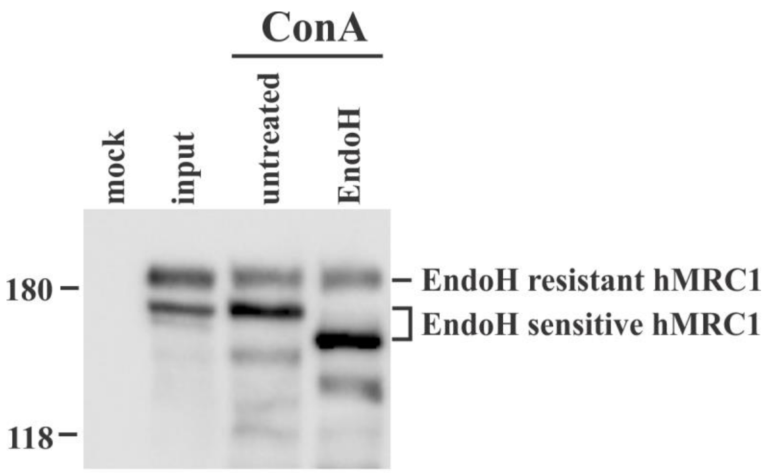

Supplemental Figure S2. Endoglycosidase H (EndoH) analysis of transiently expressed hMRC1. Transfection of HEK293T cells with pCMV6-hMRC1-HA results in the production of two bands. EndoH analysis was performed as described in Materials and Methods followed by immunoblot analysis with an antibody to the HA epitope. Mock transfected cells (lane 1) and input sample (lane 2) represent

specificity controls. ConA enriched samples (lanes 3 & 4) were either mock treated (lane 3) or were treated with EndoH enzyme (lane 4). The result confirms that the upper band represents mature (i.e. EndoH resistant) hMRC1 while the lower band represents an EndoH sensitive (i.e. immature) form of hMRC1.

V3-loop

virus isolate	aa	base / stem / crown / stem / base	aa	Net Charge	Tropism	inhibited by hMRC1
NL43	294	ctrpnnntrksiriqrgp--grafv-tigki-gnmrqahc	329	+8	X4	yes
49.5	294	ctrpnnntrksihi--gp--grafy-ttgeiigdirqahc	328	+3	R5	no
AD8	292	ctrpnnntrksihi--gp--grafy-ttgdiigdirqahc	326	+3	R5	no
SIVcpz	289	ctrlgnktiegipi--gp--gqifyrtktvv-gdtrgaec	320	+2	R5	yes
ROD10	305	ckrpgnktvkqimlmsghvfhshyq-pink---rprqawc	340	+6	X4	yes
TF #1	304	ctrphnntrksihi--gp--grawy-atgdiigdirkayc	338	+4	R5	yes
TF #2	297	ctrhnnntrksini--gp--grafy-atgkiigdirqahc	331	+5	R5	yes
TF #3	301	ctrpnnntrksihi--gp--grafy-atgniigdirkayc	335	+5	R5	yes
TF #4	295	cmrpgnntsksihm--ga--lrafh-atsriigdtrrahc	329	+5	R5	yes
TF #5	296	ctrpnnntrksiti--gp--grafy-atgdiigdirqahc	330	+3	R5	yes
TF #6	297	ctrpnnntrksihi--ap--grafy-atgeiigdirkayc	331	+4	R5	no
TF #7	304	ctrpnnntsksihm--gp--ggaff-atgriigdirkayc	338	+4	R5	no
TF #8	295	ctrpgnntrrsini--gp--grafy-atgaiigdirkahc	329	+5	R5	no

Supplemental Figure S3. Alignment of Env V3 domains of viruses used in this study. The Env region of all viruses, including eight T/F viruses was verified by sequence analysis and the V3 domains were aligned. Definition of base, stem, and crown domains of the V3-loops was adapted from Friedrich et al (40). Amino acid positions of the V3 domains within the individual Env proteins are listed at the ends. Basic amino acids (R, K) are shown in blue; negatively charged residues (D, E) are shown in red.- Dashes indicate deletions. Red boxes indicate a possible N-linked glycosylation site. The net charge of each V3 region is shown on the right. Viral tropisms and sensitivity to inhibition by hMRC1 as experimentally determined in this study are listed on the right as well.
